# Insights from veterinary models for advancing oncolytic virotherapy through comparative oncology

**DOI:** 10.3389/fmolb.2025.1615393

**Published:** 2025-05-22

**Authors:** Almohanad A. Alkayyal

**Affiliations:** Department of Medical Laboratory Technology, Faculty of Applied Medical Sciences, University of Tabuk, Tabuk, Saudi Arabia

**Keywords:** oncolytic viruses, comparative immuno-oncology, animal models, immunotherapy, veterinary oncology

## Abstract

Cancer is a complex disease affecting both humans and animals. Comparative immuno-oncology explores immune responses across species to develop effective cancer therapies. Oncolytic viruses (OVs) serve as both direct tumor-lysing agents and immune stimulants, making them an attractive therapeutic option. This review highlights the role of naturally occurring tumors in animal models for OV-based cancer immunotherapy. We examine immune responses in different species, the latest advancements in OV therapy, and the role of precision medicine in veterinary oncology. Understanding these comparative aspects enhances OV translation from preclinical to clinical applications in both veterinary and human oncology.

## 1 Introduction

Cancer poses a significant health threat globally, affecting both humans and a wide array of animal species. It is a complex disease characterized by uncontrolled cell growth and proliferation, accompanied by immune system evasion and complex interactions within the tumor microenvironment. Historically, preclinical cancer research has heavily depended on murine models artificially engineered to develop tumors (Guerin et al., 2020). However, these induced models often lack key aspects of naturally occurring cancers, such as spontaneous development, immune system interactions, and metastatic behaviors, limiting their translational utility (Onaciu et al., 2020; Cekanova and Rathore, 2014). Comparative oncology has emerged as an essential bridge connecting veterinary and human medicine by leveraging spontaneously occurring tumors in companion animals, such as dogs, cats, and horses, which share considerable genetic, biological, and environmental similarities with human cancers (Mestrinho and Santos, 2021). These animal models present unique advantages by closely mimicking the human disease trajectory, including spontaneous tumor development, immune suppression, tumor heterogeneity, and resistance to treatments, thus providing clinically relevant insights that cannot be captured entirely by induced murine models (Mestrinho and Santos, 2021). Among innovative cancer treatments, immunotherapy has dramatically transformed therapeutic paradigms in human oncology, significantly improving patient outcomes in several cancer types (Garg et al., 2024; Fan et al., 2023). However, not all patients respond equally, highlighting a need for personalized and integrative treatment strategies (Szeto and Finley, 2019; Maciejko et al., 2017). Within this therapeutic landscape, oncolytic viruses (OVs) represent a rapidly evolving approach that combines direct oncolytic activity with immune-mediated anti-tumor responses. OVs selectively infect and replicate within malignant cells, causing direct tumor cell lysis, releasing tumor-associated antigens, and consequently stimulating systemic immune responses (Lin et al., 2023). The cross-species comparative approach provided by veterinary oncology is instrumental in understanding complex interactions between tumor cells, viruses, and host immune responses (LeBlanc et al., 2025; Pinello et al., 2022). Veterinary patients often share the same environmental exposures and similar healthcare interventions as humans, making them excellent models for investigating the immune mechanisms underlying therapeutic responses to OVs and for testing novel immunotherapeutic combinations. Veterinary studies have demonstrated that species-specific immune interactions significantly influence the efficacy of OVs, underscoring the necessity for tailored viral therapies based on comparative biological insights (Hambly et al., 2023; Hampel et al., 2024; McLinden et al., 2024; Mizuno et al., 2020; Rue et al., 2015). This review explores the role of naturally occurring tumors in animal models for developing and refining OV-based therapies. It critically examines comparative immune responses across species, highlights recent advancements in OV therapies in various animal models, discusses integration with other emerging cancer immunotherapies such as immune checkpoint inhibitors (anti-PD-1, anti-PDL-1, anti-CTLA-4, etc.,.) and chimeric antigen receptor T cell therapies (CAR-T), and addresses the significant implications of precision medicine in veterinary oncology. By harnessing insights derived from comparative oncology, the field aims to improve therapeutic outcomes in veterinary practice while simultaneously accelerating the clinical translation of OV therapies into human medicine.

## 2 Naturally occurring tumors in comparative oncology

Animal models of cancer can be broadly categorized into induced and spontaneous models. Historically, induced murine models have dominated preclinical research, providing foundational knowledge but often lacking translational fidelity (Langenau et al., 2015; Sanmamed et al., 2016). Naturally occurring tumors in companion animals such as dogs, cats, and horses overcome many of these limitations by closely mimicking the natural history, biology, and progression of human cancers (Paoloni and Khanna, 2008; King, 2021; Oh and Cho, 2023). Canine osteosarcoma, lymphoma, melanoma, mammary carcinoma, and prostate carcinoma are prime examples, with significant similarities to their human counterparts concerning genetic mutations, biological behavior, and immune dynamics (Simpson et al., 2022; Irac et al., 2019; Paoloni et al., 2009; Paoloni and Khanna, 2007). For instance, canine osteosarcoma closely recapitulates key features of pediatric human osteosarcoma, including a propensity for lung metastasis, a relatively low point mutation burden, complex chromosomal rearrangements, and recurrent genetic aberrations involving critical cancer-related genes such as Tumor Protein 53 (TP53), Retinoblastoma 1 (RB1), SET Domain Containing 2 (SETD2), and components of the Phosphatidylinositol 3-Kinase (PI3K) and Mitogen-Activated Protein Kinase (MAPK) signaling pathways (Gardner et al., 2019). Canine melanoma parallels human melanoma regarding genetic mutations, tumor aggressiveness, and immune responses (Irac et al., 2019; Paoloni et al., 2009). Likewise, feline mammary carcinoma mirrors the biology and metastatic potential of human breast cancers, including hormonal receptor status and Human Epidermal growth factor Receptor 2 (HER2) expression (Oh and Cho, 2023; Soares et al., 2016; Burrai et al., 2010). Equine sarcoids, caused by bovine papillomavirus infection, closely resemble human papillomavirus-driven skin cancers, both genetically and immunologically, (Wilson and Hicks, 2016; Parkinson et al., 2024). Additionally, equine melanomas, especially prevalent in gray horses, present similar clinical challenges to human melanomas regarding therapeutic resistance and immune evasion, (Seltenhammer et al., 2004). The translational relevance of spontaneous animal tumors is further amplified by their naturally developed immune microenvironment (Vail and MacEwen, 2000). Companion animals typically experience similar environmental exposures, dietary factors, and healthcare interventions as humans. This parallel environmental exposure significantly impacts tumor biology, immune evasion mechanisms, and therapeutic response patterns, thus providing invaluable insights into the real-world effectiveness of cancer therapies. Furthermore, these naturally occurring animal tumors often exhibit immunological complexities and heterogeneity not reproducible in artificially induced murine tumors, (Tarone et al., 2019). Spontaneous cancers develop within a fully immunocompetent host, enabling the study of genuine tumor-immune interactions, immunosuppressive mechanisms, and immune escape phenomena, (Onaciu et al., 2020; Regan and Dow, 2015). The comparative approach facilitates the identification of conserved immune biomarkers, mechanisms of immune tolerance, and potential therapeutic targets, (Siel et al., 2022). The comprehensive analysis of these spontaneous tumors supports preclinical evaluation and development of novel therapies, particularly immunotherapeutic strategies such as oncolytic virotherapy. Investigations using these comparative models can accelerate therapeutic innovation, improve the predictability of treatment responses, and facilitate successful clinical translation into human oncology. Thus, naturally occurring animal tumors serve not only as relevant translational models but also offer unique opportunities to investigate novel therapeutic strategies comprehensively, effectively bridging preclinical research with human clinical oncology.

## 3 Comparative analysis of immune responses across species

Immune responses to cancer vary significantly among species, influenced by intrinsic biological differences, genetic diversity, and environmental exposures. Understanding these variances is critical for developing broadly effective and targeted cancer immunotherapies. Canine tumors closely mimic human cancers in multiple aspects, including their propensity for creating immunosuppressive tumor microenvironments (TMEs) (Siel et al., 2022; Dow et al., 1998). These microenvironments are frequently enriched with immunosuppressive populations such as regulatory T cells (Tregs), myeloid-derived suppressor cells (MDSCs), tumor-associated macrophages (TAMs), and other inhibitory cells and molecules (Dow et al., 1998; Patkar et al., 2024; Sakai et al., 2018). Regulatory T cells in canine tumors contribute to tumor immune evasion by suppressing cytotoxic T lymphocyte function and promoting tumor tolerance, a phenomenon well documented in human malignancies such as melanoma and glioblastoma (Sakai et al., 2018). Similarly, canine malignancies exhibit elevated expression of checkpoint molecules like programmed death ligand-1 (PD-L1) and cytotoxic T-lymphocyte-associated antigen-4 (CTLA-4), further impairing anti-tumor immune responses (Ariyarathna et al., 2020). In feline models, immune responses demonstrate substantial variability influenced by tumor type and host genetics. Feline mammary carcinomas frequently exhibit variable immune cell infiltration characterized predominantly by tumor-associated macrophages (TAMs), including M2 macrophages associated with immune suppression, as well as lymphocytes such as regulatory T cells (Tregs). These immune populations significantly influence tumor progression, prognosis, and therapeutic responses (Nascimento et al., 2022; Cassali et al., 2025). Additionally, Feline fibrosarcomas, particularly vaccine associated sarcomas, demonstrate a noticeable chronic inflammatory responses primarily driven by persistent adjuvant compounds (such as aluminum-based compounds) from vaccines. These adjuvants induce sustained infiltration by inflammatory cells, including macrophages and lymphocytes, that release cytokines, free radicals, and growth factors, triggering DNA damage and activating oncogenic signaling pathways such as NF-κB and COX-2. This chronic inflammatory milieu not only promotes malignant transformation but also fosters local immune tolerance, paralleling tumor-driven immune activation and evasion mechanisms seen in human cancers (Hartmann et al., 2023; Saba, 2017). Equine tumors, notably sarcoids and melanomas, provide valuable comparative perspectives due to their large size, long disease course, and clear parallels in immune modulation observed in human cancers (Pimenta et al., 2023; van der Weyden et al., 2016). Equine sarcoids, associated with bovine papillomavirus, manifest chronic immune evasion strategies, characterized by a dense infiltration of regulatory immune cells that mirror human papillomavirus-driven cancers (Parkinson et al., 2024). Equine melanomas, particularly prevalent in aging gray horses, similarly reflect human melanomas, demonstrating immunosuppressive microenvironments and variable responsiveness to immunotherapeutic interventions (Seltenhammer et al., 2004). In contrast, traditional murine cancer models, typically involving syngeneic cell lines or genetically engineered mice, frequently fail to capture the complexity and heterogeneity of immune responses present in spontaneous human and veterinary cancers. Laboratory-induced murine tumors often exhibit more homogeneous and less immunologically complex TMEs, limiting their predictive value for human clinical outcomes (de Jong and Maina, 2010). However, engineered murine models remain valuable for understanding fundamental biological processes, facilitating controlled mechanistic studies of immune interactions, tumor progression, and therapy mechanisms. Comparative immuno-oncology, therefore, significantly complements traditional research methodologies, enabling cross-species comparisons that reveal both conserved and divergent immune pathways and responses. Leveraging these comparative insights significantly informs the rational design of oncolytic virus therapies and combinatorial immunotherapy strategies, ultimately improving translational potential and clinical outcomes in veterinary and human oncology.

## 4 Oncolytic virus-based cancer immunotherapy in animal models

Oncolytic viruses (OVs) represent an innovative therapeutic approach that combines direct lysis of tumor cells through selective viral replication with systemic activation of anti-tumor immunity (Rahman and McFadden, 2021). The therapeutic potential of OVs stems from their inherent ability to specifically infect cancerous cells, sparing normal tissues due to selective viral tropism driven by molecular and immunological differences between malignant and healthy cells (Rahman and McFadden, 2021). Upon infection, OVs cause immunogenic cell death (ICD), releasing tumor antigens, damage-associated molecular patterns (DAMPs), and cytokines, which collectively activate robust local and systemic anti-tumor immune responses (Palanivelu et al., 2022; Ma et al., 2020). Animal models serve as critical translational bridges in understanding OV dynamics, safety, efficacy, and mechanisms of action across species, ultimately guiding therapeutic applications in humans.

### 4.1 Canine models

Dogs provide a uniquely advantageous model for studying OV therapy, given their spontaneous tumor development, large tumor size, genetic similarities to human cancers, and immunocompetent status (Gentschev et al., 2014; Patil et al., 2012; Sanchez et al., 2018). Recent studies utilizing canine-specific and adapted viruses have demonstrated considerable therapeutic efficacy. For instance, canine distemper virus (CDV), engineered to selectively replicate in canine tumor cells, has shown promising results in treating aggressive canine cancers such as lymphoma and osteosarcoma, causing pronounced tumor regression and improved survival rates (Sanchez et al., 2018). Furthermore, recombinant Newcastle disease virus (NDV), known for its natural oncolytic properties, has effectively induced potent immune responses against canine melanoma, a cancer notoriously resistant to traditional treatments (Numpadit et al., 2023). Additionally, adenovirus-based oncolytic virus, Canine adenovirus type 2 (CAV2), induced significant innate immune responses characterized by increased infiltration of neutrophils and macrophages into tumor sites, accompanied by a modest adaptive immune response reflected by elevated numbers of CD4^+^ and CD8^+^ T lymphocytes in both tumor tissues and peripheral blood (Martin-Carrasco et al., 2022). Studies employing canine adenovirus vectors engineered to express immunomodulatory cytokines like interleukin-12 (IL-12) or CD40 ligand have further enhanced anti-tumor immune responses and clinical outcomes in spontaneous canine malignancies (Gentschev et al., 2014). Such studies underscore the translational potential of canine models, facilitating optimization of OV dosing regimens, immune modulation strategies, and combinatorial treatment protocols.

### 4.2 Feline models

Feline models represent another valuable avenue for exploring OV therapy due to unique viral-host interactions and immune dynamics. Spontaneous feline cancers, such as vaccine-associated fibrosarcomas and mammary carcinomas, provide robust immunological models that closely resemble aggressive human cancers (Saba, 2017; Gentschev et al., 2014; McNiel, 2001). Feline-specific oncolytic viruses have been explored in preclinical studies as potential therapeutics for feline cancers. Myxoma virus, although primarily pathogenic in rabbits, has shown promising results by inducing cytopathic effects and productive viral replication in feline cancer cell cultures, suggesting potential utility in feline oncology pending further studies (Jourdier et al., 2003). Additionally, recombinant oncolytic vaccinia virus GLV-5b451 expressing anti-VEGF single-chain antibody (GLAF-2) demonstrated significant inhibition of feline mammary carcinoma growth in xenograft mouse models, reducing tumor angiogenesis and VEGF expression within tumors (Adelfinger et al., 2014)​. Maraba virus, tested as a heterologous prime-boost vaccine strategy in cats, was found to be safe and well-tolerated, triggering transient immune responses characterized by lymphoid tissue hyperplasia, but it has yet to be specifically evaluated for direct anti-tumor efficacy in feline cancer models (Hummel et al., 2017). Collectively, these studies highlight the therapeutic potential and safety profile of certain oncolytic viruses in feline oncology, emphasizing the need for further research to understand their full immunotherapeutic capabilities.

### 4.3 Equine models

Equine models, characterized by large tumor volumes, long disease courses, and immunocompetent status, uniquely contribute to OV translational research. Equine sarcoids, caused by bovine papillomavirus infection, and equine melanomas, particularly prevalent in grey horses, offer excellent platforms to study OV therapy’s clinical safety and efficacy (Rothacker et al., 2015). Equine-specific oncolytic virus therapies, including Avipoxvirus-based recombinant vector vaccine (ALVAC-fIL2), a feline interleukin-2 immunomodulator delivered via canarypox vector, have shown therapeutic potential against equine sarcoids. Intratumoral injection of ALVAC-fIL2 in horses resulted in a high overall response rate (86%) and was well-tolerated, causing minimal adverse effects limited primarily to transient local inflammation. Although responses varied in time, a significant percentage of treated horses experienced meaningful tumor regression, highlighting ALVAC-fIL2 as a safe, feasible, and cosmetically favorable treatment option for sarcoids in veterinary practice. This therapeutic approach underscores the feasibility of canarypox-based immunotherapies in equine oncology but also highlights the necessity of further controlled studies to optimize therapeutic protocols and to fully evaluate efficacy and immune mechanisms involved​ (Saba et al., 2022).

The diverse animal models utilized in OV research provide critical comparative insights into host-virus interactions, species-specific immune responses, therapeutic safety, and long-term immunological outcomes. Such comparative studies underscore the importance of employing diverse animal models in optimizing OV treatments, ultimately enhancing their translational relevance and potential therapeutic applications across veterinary and human medicine.

## 5 Challenges in translating animal OV research to clinical applications

Despite promising advancements in oncolytic virus (OV) research through animal models, numerous obstacles impede seamless translation into clinical applications, particularly in human medicine. One primary challenge is species-specific viral tropism, as viruses optimized for one species may exhibit reduced infectivity or altered replication profiles in another. The selective replication of OVs depends critically on cellular receptors and intracellular signaling pathways, which can vary significantly across species. Hence, achieving cross-species compatibility or developing tailored species-specific OVs poses substantial technical and regulatory hurdles.

Immune-mediated viral clearance represents another significant barrier. Upon administration, the host’s innate immune system rapidly identifies and neutralizes OVs through pre-existing antiviral responses and neutralizing antibodies, limiting therapeutic effectiveness and requiring repeated or higher-dose administrations. This immune-mediated clearance not only reduces therapeutic efficacy but also presents potential safety concerns related to systemic inflammation or adverse immune reactions. Addressing this challenge involves advanced viral engineering, including modifications to viral envelopes, capsid proteins, or genomic sequences to evade immune detection or to prolong viral persistence in tumor tissues.

Optimal dosing strategies for OVs remain elusive, with considerable variations depending on tumor type, stage, and host immune status. Veterinary clinical trials highlight dosing complexities, revealing that dosing schedules optimal for canine patients might differ significantly from those effective in humans or other animals. Establishing standardized dosing protocols requires comprehensive pharmacokinetic and pharmacodynamic analyses across multiple species, ensuring consistent therapeutic outcomes while minimizing toxicities.

Regulatory frameworks in veterinary medicine further complicate the clinical translation of OV therapies. Regulatory agencies typically demand extensive safety, efficacy, and quality data, presenting logistical and financial barriers for veterinary clinical trials. Unlike human clinical trials, veterinary trials may have limited resources and logistical support, restricting their scale, scope, and generalizability. Moreover, regulatory approval processes vary considerably across jurisdictions, complicating multicenter and international studies essential for generating robust and widely applicable data.

Addressing these translational challenges requires interdisciplinary collaboration among veterinary oncologists, virologists, immunologists, regulatory authorities, and human oncologists. Future efforts must prioritize optimizing viral engineering, enhancing therapeutic synergy through combination treatments, improving clinical trial designs, and streamlining regulatory processes. Successfully navigating these complexities can significantly accelerate the translation of OV therapies from veterinary to human oncology, maximizing clinical benefits across species.

## 6 Future directions

Future research in OV therapy must strategically focus on overcoming existing translational barriers and enhancing clinical applicability across species. Continued refinement of OV strategies through advanced viral vector engineering remains a priority. Innovations such as genetic editing; clustered regularly interspaced short palindromic repeats cas9 (CRISPR-Cas9) and targeted gene insertion offer precise control over OV selectivity, potency, immune modulation, and safety profiles. Additionally, engineered OVs capable of expressing immunomodulatory cytokines, checkpoint inhibitors, or tumor-specific antigens could further potentiate therapeutic outcomes and reduce immune-mediated clearance.

Deeper investigation into host-virus interactions will significantly enhance therapeutic predictability and efficacy. Detailed comparative immunological studies elucidating the innate and adaptive immune mechanisms in response to OVs across different animal species are necessary. Such comparative insights will help to design species-appropriate immune modulatory strategies, enhancing OV efficacy and longevity in tumor tissues.

Standardization of clinical protocols and endpoints across veterinary trials is crucial for generating reproducible and comparable data. Establishing consistent dosing regimens, administration routes, and outcome assessments across multi-center veterinary clinical trials would significantly enhance data reliability and facilitate meta-analyses, thereby accelerating regulatory approvals and clinical adoption.

Exploring innovative combination therapies remains pivotal. Integration of OV therapy with immune checkpoint blockade, adoptive cell transfer therapies (CAR-T cells), targeted therapies, and conventional chemotherapeutics holds significant potential. Veterinary models offer ideal platforms for testing these complex combination strategies due to their immunocompetent nature, genetic diversity, and spontaneous cancer progression, providing crucial preclinical insights and safety profiles before human translation.

Expansion of comparative oncology research, including broad, multi-center veterinary clinical trials, is essential for bridging gaps between preclinical findings and clinical practice. Collaborative networks involving academic institutions, veterinary practices, industry partners, and regulatory bodies will foster comprehensive translational research frameworks, accelerating therapeutic advancements in both veterinary and human oncology.

## 7 Conclusion

Oncolytic virus therapies have emerged as a highly promising frontier in cancer immunotherapy, enriched significantly by insights derived from comparative oncology. Naturally occurring tumors in animal models provide robust and clinically relevant platforms, enhancing our understanding of OV biology, immune responses, and translational feasibility. Comprehensive comparative analyses across canine, feline, murine, and equine models facilitate identification of universal and species-specific therapeutic challenges, enabling precise tailoring of therapeutic strategies.

While significant progress has been achieved, ongoing challenges in viral tropism, immune clearance, dosing standardization, and regulatory complexities require sustained research efforts. Strategic refinement of viral engineering, standardization of clinical protocols, deeper investigation of immune mechanisms, and innovative combination approaches will be pivotal in overcoming current translational barriers.

Ultimately, comparative oncology remains instrumental in advancing OV therapies, offering invaluable insights and methodologies that substantially benefit veterinary patients while accelerating clinical translation for human oncology. Continued integration of veterinary and human oncology perspectives promises substantial improvements in therapeutic outcomes, precision medicine strategies, and patient survival across species.
